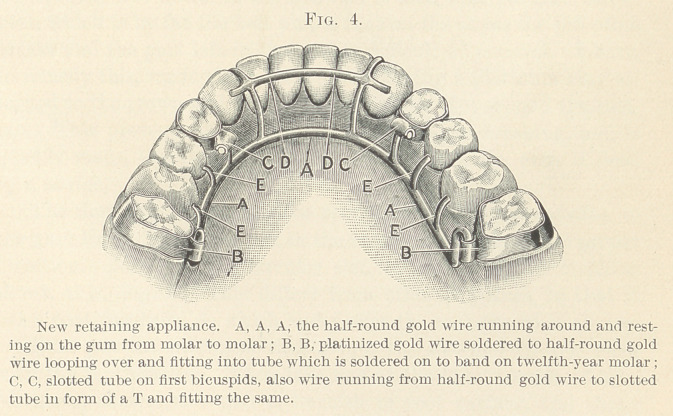# Some New Appliances

**Published:** 1901-06

**Authors:** Harry F. Hamilton

**Affiliations:** Boston, Mass.


### SOME NEW APPLIANCES.[1]

BY HARRY F. HAMILTON, D.M.D., BOSTON, MASS.

### A NEW TOOTH-BRUSH.

MANY years ago I had my attention called to injuries to the gums and teeth caused by excessive use of the tooth-brush. I have since then observed carefully all the cases which came under my care, and have seen many where the teeth have been practically ruined and their usefulness destroyed years before they should have been merely by the conscientiousness of the patients in trying to keep them clean and guard against caries.

I have seen the gums brushed away over a quarter of an inch in three months, and come down over the places again upon the brushing being stopped. In many cases the gums have come down three-sixteenths of an inch on prohibiting all brushing for a limited time.

Unless one observes carefully, and takes impressions and measurements, these injuries are not thought of, except as a natural recession, but if the dentist will think how few of these sharp loaf-sugar-shaped recessions are to be seen in the mouths of those never using the brush, he will realize what a danger it can be in willing but unskilful hands.

The gum over the left superior cuspid is the first to go, then follows the left superior and inferior bicuspids and the right superior cuspid, and then the left superior lateral, and so on through the whole mouth. The right superior lateral is not harmed until about the last, as it is not reached easily by a brush in the right hand. Later the gums in the interproximal spaces give way from the friction and from losing the support on the labial surfaces, and we have food crowding in and causing decay. It is at this time the

---

[1] Read before the American Academy of Dental Science, March 6, 1901.

patient complains of the amount of work to be done, as he has always brushed his teeth many times a day.

A general irritability is set up by the vigorous friction, so the patient is not comfortable unless he has had his scrub after every meal. Patients always assure you they brush carefully, often saying they brush up and down and not across, but the marks are there and cannot be explained away. In many cases I have had to prohibit the use of the brush altogether, a thankless task until the patient becomes interested in the gradual covering of the unsightly places.

The gum is soft and spongy at first, but gradually hardens to its proper density. To remedy this abuse I tried prescribing the badger-hair brush. With a few persons this is a success, but it must be used without pressure, as most persons bear on hard and the hairs part in the centre and become merely a mat. To avoid this I have had the brush made which I submit for your inspection. It has alternate tufts of badger-hair and bristles, the latter being considerably shorter, their office being to give firmness and support to the badger-hair tufts. (The badger-hair tufts are shown at A, the bristle tufts at B, Fig. 1.)

The brush is not intended for general use, but for those patients who have this tooth-brush recession, or are in a way to have it. It should be supplemented by the use of a cloth over the end of the finger, with powder, once or twice a week.

I find it difficult to coax the gums down in patients over forty, although occasionally the results are strikingly good, but in younger patients I have had more pleasure in seeing the change I have brought about than in almost any other of my dental efforts.

### A VALUABLE NEW DEVICE TO RELIEVE AN ABSCESS.

I submit to the Academy a device which has been of considerable use to me for some three years, and one that wins the immediate gratitude of the patient. It started by my slipping the end of the rubber tube of my saliva ejector over the cavity of a tooth, which had begun to ulcerate, and where I had opened into the pulp-chamber. The tooth was aching fiercely. Probably in a few hours it would have been all right, but I put on my rubber tube, started the ejector, and in two minutes the pain was gone. Since then I have used it frequently to relieve pressure, and also to get pus from a blind abscess. I use a heavy tube, as the ordinary one flattens by

Fig. 1.

Tooth-brush. A, badger-hair; B, bristle.

Fig. 2.

Abscess ejector.

Fig. 3.

Improved crown.

Fig. 4.

New retaining appliance. A, A, A, the half-round gold wire running around and resting on the gum from molar to molar; B, B, platinized gold wire soldered to half-round gold wire looping over and fitting into tube which is soldered on to band on twelfth-year molar; C, C, slotted tube on first bicuspids, also wire running from half-round gold wire to slotted tube in form of a T and fitting the same.

the suction. An ordinary rubber tube, hard by age, will do if not too rotten.

The tips which I have made to facilitate the suction are metal tubes sliding into the end of the rubber tube, and having a concave disk (B, Fig. 2) soldered a quarter of an inch from the end. This disk is filled with soft gutta-percha filling, heated and forced into the cavity, making a tight joint.

I show this attached to a bicycle pump, which can be used for an exhaust pump by reversing the valve. This can be used where the saliva ejector has not power enough. I have also used this exhaust in cases where I wished to draw a small quantity of blood and a leech was not at hand. A cut with the trephine lance, the rubber tube applied to the gum, and a quantity of blood is taken. The end of the tube should be small for this, as a large one is painful.

### AN IMPROVED CROWN.

The painful trimming of the root, fitting, and subsequent recession of the gums in a Richmond crown have condemned it for my use. The weakening of the root by the large pin, and the large quantity of cement necessary in the Logan crown, likewise forbids its use, where the best is desired, and my experience is that some modification of the Bonwill crown is more satisfactory than any other to dentist and patient.

I much prefer setting the pin in the root first, rather than in the crown. The defect in the Bonwill, if used with the How screw-post, which is by far the best pin, is that on the stress the pin often breaks and the part left in the root is difficult of removal. I have, to remedy this, made and used a crown having a platinum ring attached to the crown and fitting into a circular slot around the root-pin. This prevents the root splitting or the pin breaking, and is quickly made, everywhere fitting the root accurately, even if done by a novice.

The first step is to shape the root saddle-shape, conforming to the gum line. Next set a pin in the root and bend it in the proper direction. Next cut the circular slot, by means of the trephine shown at *a*, Fig. 3. This differs from other trephines, in that it has a centring tube, A, which slides over the root-pin when in use.

Next put the platinum ring *d* into the slot and trim it flush with the root surface. Then take one of the blanks made of thin platinum, shown at *b* and *c*, burnish to root surface, and shape to come exactly to the circumference of the root, as shown at *e*, and cut the tube off even with the pin. Put a little sticky wax on the under side, heat it, press in place, and cool. The band then comes off attached to the blank. Invest in some quick hardening investment and solder with pure gold. Return to the tooth, take an impression over it, and finish by the model, which consists in fitting roughly a cross-pin tooth, bending the pins to nip the tube, so as to hold while the body is packed to the shape required and baked. The finished crown ready for cementing is shown at *f*, Fig. 3.

## Figures and Tables

**Fig. 1. f1:**
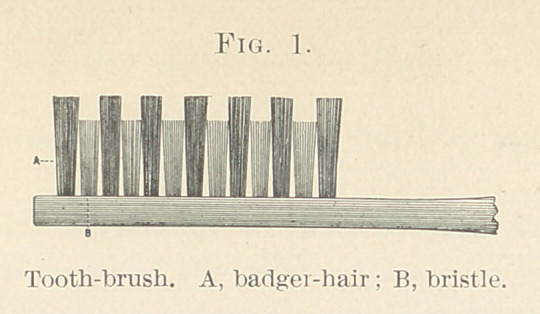


**Fig. 2. f2:**
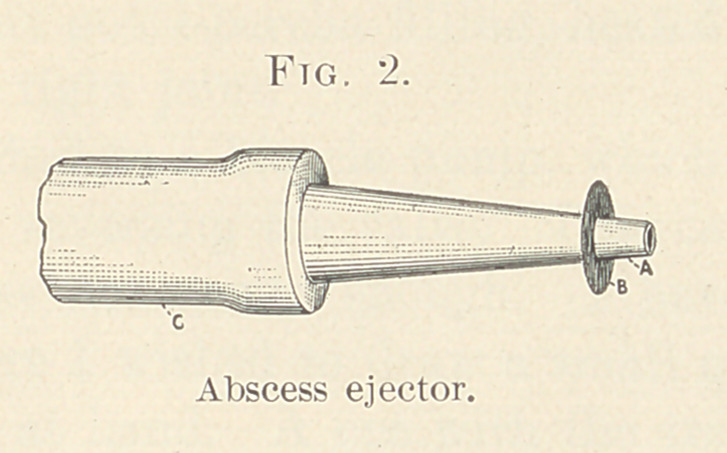


**Fig. 3. f3:**
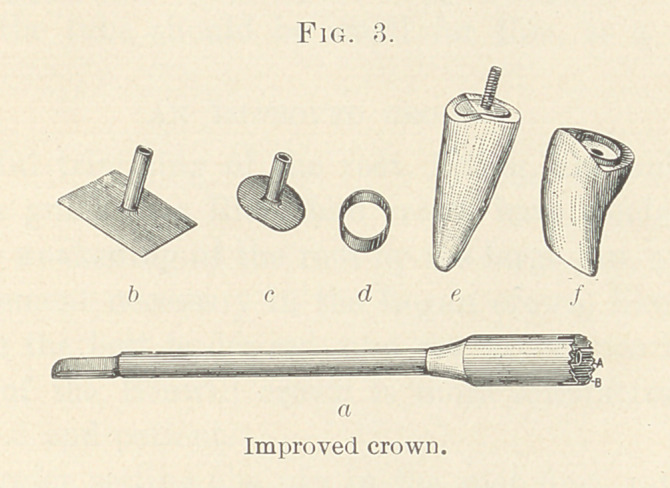


**Fig. 4. f4:**